# UV light-mediated corneal crosslinking as (lymph)angioregressive pretreatment to promote graft survival after subsequent high-risk corneal transplantation (CrossCornealVision): protocol for a multicenter, randomized controlled trial

**DOI:** 10.1186/s13063-024-08011-1

**Published:** 2024-03-06

**Authors:** Johanna Wiedemann, Deniz Hos, Endrik Limburg, Ulrike Zettelmeyer, Petra Schiller, Jeremy Franklin, Björn Bachmann, Daniel Böhringer, Tina Dietrich-Ntoukas, Thomas A. Fuchsluger, Gerd Geerling, Stefan J. Lang, Wolfgang Johann Mayer, Siegfried Priglinger, Thomas Reinhard, Berthold Seitz, Claus Cursiefen

**Affiliations:** 1 Department of Ophthalmology, University Hospital Cologne, Cologne, Germany; 2 Clinical Trials Centre of Cologne (CTCC), University of Cologne, Cologne, Germany; 3 Institute of Medical Statistics and Computational Biology (IMSB), Faculty of Medicine, University of Cologne, Cologne, Germany; 4 Department of Ophthalmology, Charité – Universitaetsmedizin Berlin, corporate member of Freie Universitaet Berlin, Humboldt Universitaet zu Berlin, and Berlin Institute of Health, Berlin, Germany; 5 Department of Ophthalmology, University of Rostock, Rostock, Germany; 6 Department of Ophthalmology, University of Düsseldorf, Düsseldorf, Germany; 7 Department of Ophthalmology, University of Freiburg, Freiburg, Germany; 8 Department of Ophthalmology, LMU, University of Munich, Munich, Germany; 9 Department of Ophthalmology, Saarland University Medical Center, Homburg/Saar, Germany; 10 CECAD Cluster of Excellence, University of Cologne, Cologne, Germany

**Keywords:** Cornea, (Lymph)angioregressive preconditioning, Multicenter randomized clinical trial (RCT), Corneal transplantation, Crosslinking (CXL), Graft rejection, Neovascularization, High-risk corneal transplantation, Randomized controlled trial

## Abstract

**Background:**

Good vision highly depends on the transparency of the cornea, which is the “windscreen” of the eye. In fact, corneal blindness due to transparency loss is the second most common cause of blindness worldwide, and corneal transplantation is the main cure. Importantly, the cornea is normally avascular but can secondarily be invaded by pathological (blood and lymphatic) vessels due to severe inflammation, and the survival prognosis of a corneal graft mainly depends on the preoperative vascular condition of the recipient’s cornea. Whereas transplants placed into avascular recipient beds enjoy long-term survival rates of > 90%, survival rates significantly decrease in pathologically pre-vascularized, so-called high-risk recipients, which account for around 10% of all performed transplants in Germany and > 75% in lower and middle-income countries worldwide.

**Methods:**

This parallel-grouped, open-randomized, multicenter, prospective controlled exploratory investigator-initiated trial (IIT) intends to improve graft survival by preconditioning pathologically vascularized recipient corneas by (lymph)angioregressive treatment before high-risk corneal transplantation. For this purpose, corneal crosslinking (CXL) will be used, which has been shown to potently regress corneal blood and lymphatic vessels. Prior to transplantation, patients will be randomized into 2 groups: (1) CXL (intervention) or (2) no pretreatment (control). CXL will be repeated once if insufficient reduction of corneal neovascularization should be observed. All patients (both groups) will then undergo corneal transplantation. In the intervention group, remaining blood vessels will be additionally regressed using fine needle diathermy (on the day of transplantation). Afterwards, the incidence of graft rejection episodes will be evaluated for 24 months (primary endpoint). Overall graft survival, as well as regression of corneal vessels and/or recurrence, among other factors, will be analyzed (secondary endpoints).

**Discussion:**

Based on preclinical and early pilot clinical evidence, we want to test the novel concept of temporary (lymph)angioregressive pretreatment of high-risk eyes by CXL to promote subsequent corneal graft survival. So far, there is no evidence-based approach to reliably improve graft survival in the high-risk corneal transplantation setting available in clinical routine. If successful, this approach will be the first to promote graft survival in high-risk transplants. It will significantly improve vision and quality of life in patients suffering from corneal blindness.

**Trial registration:**

ClinicalTrials.gov NCT05870566. Registered on 22 May 2023.

## Administrative information

**Table:** 

**Title {1}**	UV light-mediated corneal crosslinking as (lymph) angioregressive pretreatment to promote graft survival after subsequent high-risk corneal transplantation: protocol for a multicenter, open-randomized controlled (CrossCornealVision)
**Trial registration {2a and 2b}.**	Clinical Trials.gov: NCT05870566. Registered on 22. May 2023.
**Protocol version {3}**	Version of 15.09.2023, Version V03_0
**Funding {4}**	Bundesministerium für Bildung und Forschung (BMBF, grant no: 01KG2127)
**Author details {5a}**	Department of Ophthalmology, University Hospital Cologne, Cologne, Germany
**Name and contact information for the trial sponsor {5b}**	University of Cologne, represented by Prof. Dr. med. Claus Cursiefen, Department of Ophthalmology, University Hospital Cologne, Kerpener Str. 62, 50937 Cologne, Germany
**Role of sponsor {5c}**	The sponsor is responsible for financing, conducting and supervision of the trial, including quality management, trial design, data handling (Trials Centre Cologne (CTCC) at the University of Cologne (Gleueler Str. 269, 50935 Cologne)), investigator selection as well as data analysis.

## Introduction

### Background and rationale {6a}

The main treatment of corneal blindness is corneal transplantation, which is the most commonly performed type of transplantation, with more than 50,000 surgeries per year in Europe and about 9000 in Germany. Out of these, about 10% in Germany and more than 75% in lower and middle-income countries are so-called high-risk full thickness penetrating keratoplasties (pKPLs) with pathological corneal neovascularization (CoNV) including blood and lymphatic vessels in the recipient bed [1–5]. The ingrowth of CoNV into the normally avascular cornea abrogates the immune-privileged state of the cornea and significantly impairs graft survival [6–8].

Thus, transplant rejection rates depend on the degree of recipient CoNV and reach more than 50% in the first two years in high-risk eyes [6–9]. Rejection is the main cause of graft failure, leading to the recurrence of corneal opacification, loss of quality of life, inability to work or live independently, additional costs, and blindness. So far, no established approach reliably improves graft survival in high-risk patients, including Human leukocyte antigen (HLA)-typing or systemic immunosuppression. Therefore, an effective therapy to reduce the risk of corneal graft rejection and thereby improve graft survival would alleviate the disease burden and improve the quality of life of numerous patients.

Previously, we and others have shown that lymphatic vessels are even more important than blood vessels in mediating immune responses after high-risk corneal transplantation [10, 11]. In addition, using the murine model of high-risk corneal transplantation, we have recently demonstrated that preoperative regression of pathologic corneal blood and lymphatic vessels before transplantation (“(Lymph)angioregressive Preconditioning”) significantly improves graft survival [11–13].

We also demonstrated that corneal crosslinking (CXL) with Riboflavin as a photosensitizer, a technique routinely applied in the clinic to stabilize corneas, e.g., in keratoconus, leads to the regression of CoNV in the experimental setting and patient eyes [13–15]. Importantly, graft survival was significantly improved when subsequent corneal transplantation was performed in mice with previous CXL and in early clinical case reports [13, 14]. All previous studies, however, were experimentally or retrospective without controls and with small sample sizes. Our ITT aims to test the effectiveness of pretransplant (lymph)angioregression by CXL in improving graft survival in high-risk corneal transplant patients. This is the first prospective clinical trial in ophthalmology and transplant medicine in general concerning [lymph]angioregressive preconditioning of a transplant recipient site. Positive results could also benefit other areas of transplantation immunology [16, 17].

### Objectives {7}

The trial's primary objective is to prove and estimate the reduction of graft rejection rates and subsequent graft failure rates after corneal transplantation in vascularized high-risk eyes by preoperative (lymph)angioregressive pretreatment using CXL. Secondary objectives are functional graft survival rate, rejection-related graft failure rate, regression of pathological corneal vessels after CXL (after 1^st^ and eventually 2^nd^), number of CXL needed for successful regression > 50% of CoNV, recurrence of CoNV after CXL and after transplantation and visual acuity. Additional secondary endpoints are vision-related quality of life and safety.

### Trial design {8}

This trial is a parallel-grouped, open-randomized, multicenter, controlled exploratory ITT. Groups (intervention group [once or twice CXL] and control group [no CXL]) will be randomly allocated in a 5:4 ratio. One hundred and ten participants will be included in the trial (intervention group 61 patients, control group 49 patients).

### Participants

#### Study setting {9}

This multicenter clinical ITT will be conducted at seven sites in Germany (Berlin, Cologne, Duesseldorf, Freiburg, Homburg/Saar, Munich, and Rostock). If necessary, further qualified trial sites may be recruited for the trial. The listing of trial sites, principal investigators and investigators, and further trial staff will be kept and continuously updated on our homepage (http://augenklinik.uk-koeln.de/forschung/klinische-studien/). A final version of this list will be attached to the trial's final report.

#### Eligibility criteria {10}

Eligibility for patients: Male and female patients aged ≥ 18 years with pre-vascularized corneas ≥ 2 corneal quadrants with the need for corneal transplantation due to central corneal opacification and low visual acuity due to this condition can be included in the study.

Patients who qualify for the study and want to participate must sign a written informed consent (IC) before inclusion. Exclusion criteria include certain eye conditions (dysregulated glaucoma, active uveitis, or corneal ulcer), allergies, contraindications to medication or surgery, significant medical conditions, and inability to comply with the study protocol.

Eligibility for study centers and surgeons: All study centers must have at least one expert corneal surgeon. To ensure follow-ups and if repetition of CXL is needed, another ophthalmologist will be involved.

#### Who will take informed consent? {26a}

After completion of screening at visit one, patients will be informed verbally and in writing incomprehensible language about the nature, scope, and possible consequences of the surgery, the possible pretreatment with CXL, and the randomization by the local principal investigator/investigator. Patients will sign the IC to the trial, where all potential interventions and the randomization will be discussed before inclusion in the trial, as well as an IC form specific to keratoplasty at least 24 h before the pKPL operating day. All questions will be addressed.

The participant information materials and informed consent forms are available from the corresponding author on request. The signed consent form is archived as original in the investigator site file at the trial site. Trial patients receive copies of the written information sheet and the signed IC form.

#### Additional consent provisions for collection and use of participant data and biological specimens {26b}

So far, additional data or biological specimen sampling and analysis are not planned; however, this might change in the future. In case of any changes in the study design, the EC and renewed IC approval is required.

## Interventions and outcomes

### Explanation for the choice of comparators {6b}

Parallel groups will be randomly allocated to “CXL” versus “no CXL” before corneal transplantation in a 5:4 ratio; “no CXL” before corneal transplantation is the current standard treatment. There is no evidence-based, standardized, valid clinical method to improve corneal transplant outcome in high-risk patients. We chose CXL to precondition the recipients’ cornea as it has shown high efficacity in regressing CoNV and barely any side effects [14]. A sham CXL procedure will not be performed in this study.

### Intervention description {11a}

pKPL replaces all layers of the diseased central cornea with an allogeneic healthy graft from a deceased donor. pKPL will be performed as a standard full-thickness penetrating procedure, and the graft (individualized size between 6.5 and 8.25 mm in diameter) will be secured with at least 16 interrupted (or double running star-shaped) 10-0 nylon sutures.

CXL is a corneal treatment involving riboflavin and irradiating the cornea with UV-A light. An accelerated protocol is used, which involves higher illumination intensity (9 mW/cm^2^) and shorter UV-A irradiation time (10 min) [18]. A shield protects the limbus during the procedure to prevent damage to the limbal stem cells as we plan to irradiate the complete cornea. CXL will be performed in the intervention group to stabilize the recipient cornea and to reduce CoNV 8 to 10 weeks before pKPL. The control group will undergo pKPL without prior CXL. CXL can be repeated once at least 14 days before transplantation if CoNV is not completely regressed.

In case of residual CoNV on the day of transplantation, fine needle diathermy will be used in the intervention group. It involves coagulating blood vessels using a monopolar cautery unit and a bent needle. In the control group, no fine needle diathermy will be performed.

Postoperatively, all patients will be treated with topical corticosteroids and antibiotics, and a systemic carboanhydrase inhibitor will be given to reduce intraocular pressure on the transplant.

### Criteria for discontinuing or modifying allocated interventions {11b}

Drop-out or early discontinuation indicates a patient who has signed an IC, was eligible and enrolled with at least one trial-specific assessment, and discontinued the clinical trial for any reason before the end of the trial, as defined in the trial protocol. Reasons for drop-out/discontinuation are defined as withdrawal upon participant´s request; investigator-initiated discontinuation (e.g., medical reasons); lost to follow-up: cessation of participation without notice or action by the patient; sponsor-initiated discontinuation. Reasons for sponsor-initiated discontinuation may be insufficient regression of CoNV or prolonged healing phase after CXL so that the timeframe to the pKPL can not be respected.

### Strategies to improve adherence to interventions {11c}

Participants will receive a detailed, written treatment plan from clinical or study nurses explaining how and when to take their medications to encourage and monitor adherence. Participants who cannot apply eye drops themselves will receive treatment from the clinical nurses during the hospital stay. Attrition bias will be minimized by sending timely reminders of follow-up appointments to patients and arranging substitutes for any missed visits.

In general, corneal transplant patients have a high adherence to treatment as they usually already have a long ophthalmologic history and are eager to improve the outcome of their transplant. The allocation rate of 5:4 was chosen to allow a (slightly) higher number of patients in the intervention group since CXL is a minimally invasive and safe approach expected to reduce the risk of transplant rejection and discover even rare complications.

### Relevant concomitant care permitted or prohibited during the trial {11 d}

There are no restrictions on local and systemic therapies for patients receiving keratoplasty, including immunosuppressants. Topical steroids will be used postoperatively, with monthly reduction, topical antibiotics for two weeks, and cycloplegia during the first week. Systemic carboanhydrase inhibitors will reduce pressure on the transplant, and topical steroids and antibiotics are used and tapered as needed. Patients with corneal pathology due to herpesvirus will be treated according to guidelines using broad antivirals.

### Provisions for post-trial care {30}

All patients will be followed up for 2 years after corneal transplantation. After the end of the trial, further treatment will be based on the respective clinical findings and solely determined by the responsible ophthalmologist. For all trial subjects enrolled travel accident insurance is provided.

### Outcomes {12}

Primary endpoint: The primary endpoint is the time to the first corneal endothelial graft rejection episode within 24 months after transplantation.

Secondary endpoints: Secondary endpoints are overall functional graft survival rate, rejection-related graft failure rate, regression of pathological corneal vessels after CXL (after 1st and eventually 2nd), number of CXL needed for successful regression > 50% of CONV, recurrence of CoNV after CXL and after transplantation, visual acuity (using ETDRS charts; assessment before CXL, before transplantation and 3, 6, 12, 18, and 24 months after transplantation). Additional secondary endpoints are the vision-related quality of life measured using the National Eye Institute Visual Function Questionnaire (NEI-VFQ25) and the safety, organizational, and administrative aspects of the trial [19, 20].

The anterior segment and the cornea will be examined by slit-lamp biomicroscopy and photo-documented. The corneal thickness will be documented by pachymetry. Visual acuity will be measured by ETDRS charts, and vision-related quality of life will be measured using the NEI-VFQ25 [19, 20].

### Participant timeline {13}

The planned participant timeline over the whole trial duration is shown in Table 1.

**Table 1:** Timeline over the trial duration

First patient first visit (FPFV):	Quarter (Q) IV 2023
Last patient first visit (LPFV):	Q III 2025
Last patient last visit (LPLV):	Q III 2027
End of trial:	Q IV 2027
Final study report:	Q III 2028

**Fig. 1:**
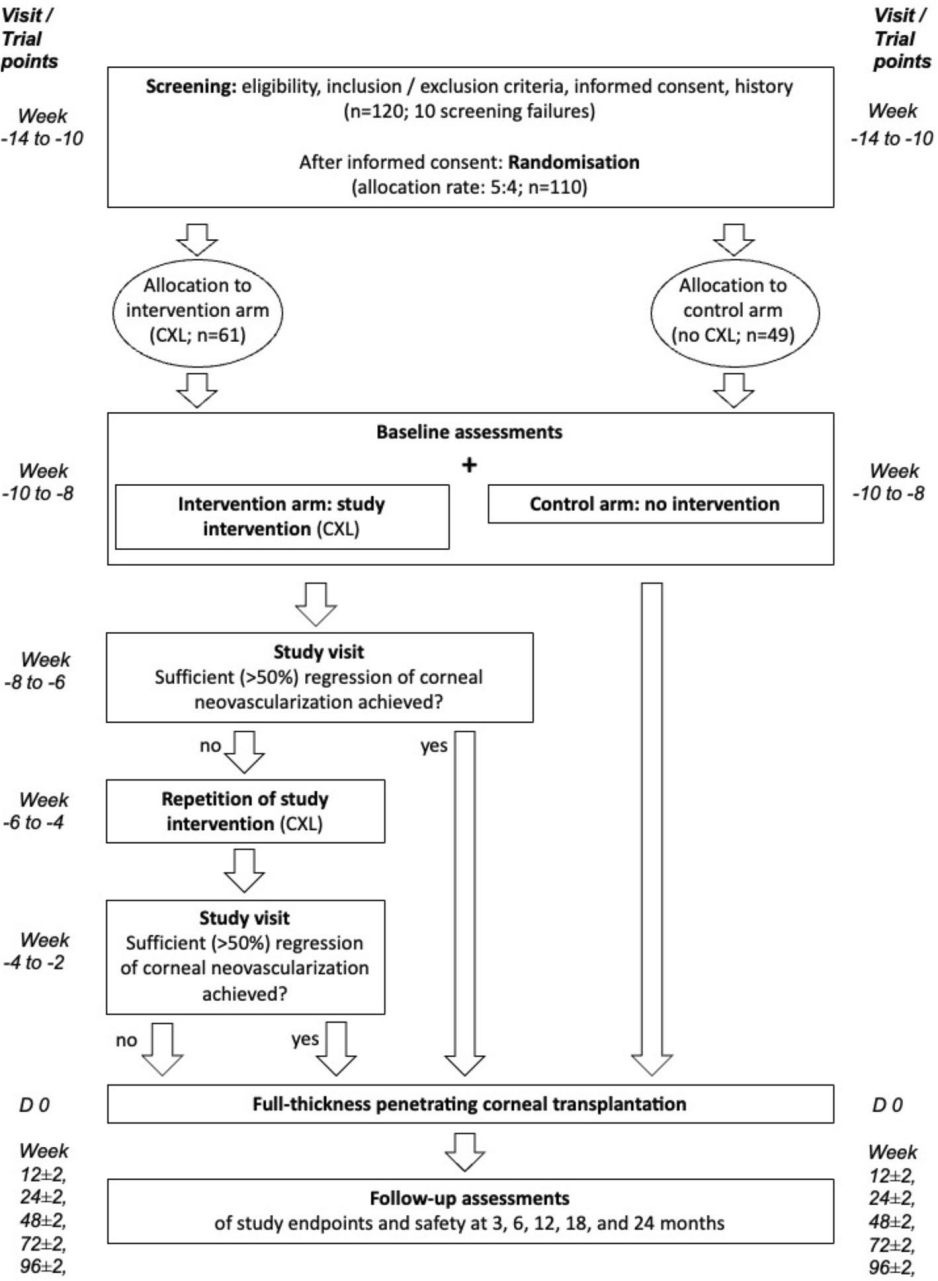
Flowchart of the trial

The planned participant timeline of visits with procedures per visit and overview of the trial as a flowchart is shown in Table 2 and Fig. 1.

**Table 2:** Participant timeline of visits with procedures

Visits	Screening	Baseline	Control after CXL^a^	Repeat study intervention^b^	Control after the second CXL	Transplantation^c^	FU				
Trial points	Week −14 to −10	Week −10 to −8	Week −8 to −6	Week −6 to −4	Week −4 to −2	D 0	Week 12±2 wk	Week 24±2 wk	Week 48±2 wk	Week 72±2 wk	Week 96±2 wk
Eligibility and history	X										
Informed consent	X										
In-/exclusion criteria	X										
Demographics	X										
Medical history	X										
Slit lamp exam	X	X	X		X		X	X	X	X	X
Standardized digital photo		X	X		X		X	X	X	X	X
Laser-FlareCell-Meter^d^		X					X	X	X	X	X
Corneal tomography	X	X	X		X		X	X	X	X	X
SL-OCT	X	X	X		X		X	X	X	X	X
Visual acuity	X	X	X		X		X	X	X	X	X
Concomitant medication	X	X	X		X		X	X	X	X	X
Randomization^e^	X										
Vision-related quality of life		X					X	X	X	X	X
CXL (intervention group only)		X		X							
Transplantation						X					
AE review and evaluation			X	X	X		X	X	X	X	X
Corneal endothelial count							X	X	X	X	X
Macula OCT^f^	X						X	X	X	X	X

*AE *Adverse event, *CXL *Corneal crosslinking, *FU *Follow-up, *EOT *End of trial, *SL-OCT *Slit lamp optical coherence tomography

^a^Two weeks after the first CXL, ^b^when regression of corneal neovascularization is < 50%, repeat CXL 4 weeks after first CXL, ^c^depending on availability of the graft and randomization, transplantation will take place between 8 and 10 weeks after baseline visit, ^d^optional (if available), ^e^after obtaining IC and completion of screening, ^f^if deemed necessary by the investigator

### Sample size {14}

Sample size calculation is based on the primary endpoint, time from surgery to the first graft rejection episode, comparing the intervention and control groups using the log-rank test. The expected rejection rate in the control group was estimated using rates given in previously published studies [6, 21]. An overall 2-year rejection rate of 50% was assumed for the control group. The study was powered to detect a reduction in the 2-year rejection rate from 50 to 25% (hazard ratio 0.42) with 80% power at a significance level of 0.05 (assuming exponential survival curves and accrual of 22 months, with a subsequent follow-up period of at least 24 months). An allocation rate of 5:4 and continuous loss-to-follow-up cumulating to 20% at 2 years was accounted for, yielding a total sample size of 110 (intervention group: *n* = 61, control group: *n* = 49). Calculations were performed using SAS 9.4 (Proc Power).

### Recruitment {15}

Participants will be recruited among patients visiting the outpatient clinic or from the corneal transplantation waiting lists. Patients will receive a detailed explanation of the risks and benefits of the trial to ensure that they fully understand the specifics of the study. Then they will be asked to provide a signed IC form voluntarily. The organizers will try to reduce the loss of participants by reminding them of their appointments and rescheduling if needed.

### Assignment of interventions: allocation

#### Sequence generation {16a}

After signed IC, patients are randomly assigned to CXL (intervention group) or no CXL (control group) using the 24-7 Internet online randomization tool ALEA Clinical (FormsVision BV, Abcoude, NL). Randomization is stratified by the center and surgeon. A statistician, independent of the trial team, generated the randomization list using permuted blocks of varying sizes and uploaded it in ALEA. The study will compare the effects of CXL versus no CXL before corneal transplantation in high-risk patients with CoNV. The participants will be assigned to either group by chance, using a 5:4 ratio and a web-based service that ensures fair and unbiased allocation.

#### Concealment mechanism {16b}

Randomization is implemented by central 24-7 Internet randomisation service ALEA. Complete blinding is not possible (see {17a}). The ALEA service is accessed via a validated password website to ensure concealment.

#### Implementation {16c}

The study physician of the respective participating center will enroll eligible participants and randomize them via ALEA. Independent randomization administrators implement and manage the trial in ALEA and set system roles and access rights. Therefore, the allocation sequence is not accessible by investigators managing or assessing the study participants and the randomization result is unpredictable. The study physicians who decide whether a repetition of CXL is necessary are not the same as the surgeons.

### Assignment of interventions: blinding

#### Who will be blinded {17a}

This exploratory trial does not involve a sham CXL procedure, so concealing patients’ group assignment to surgeons is impossible. The clinical signs of treatment or no treatment should be easily identifiable. In case of successful preconditioning, there would be vessel regression, ghost vessels, and maybe even a small regression of corneal opacification. However, to minimize the risk of performance and detection bias, we will blind the surgeons and examiners of physician-reported outcomes and endpoints as much as possible. This means that the surgeon will not be the one to decide whether CoNV regressed sufficiently and if additional reducing treatment is required. Graders from the Cologne Ophthalmological Reading and Image Analysis Center (CORIC) will independently confirm the diagnosis of rejection episodes and graft failure and quantify CoNV changes.

#### Procedure for unblinding if needed {17b}

N/A. No blinding is planned (see above). Thus, there is no procedure for unblinding.

### Data collection and management

#### Plans for assessment and collection of outcomes {18a}

Administrative trial project management, data management, monitoring, and safety management are done by the Clinical Trials Center Cologne (ZKS). Potential patients will be pre-selected when encountered during clinical routine or by viewing the corneal transplantation waiting lists by the study physicians. After screening assessments are completed, inclusion and exclusion criteria will be validated. If all inclusion criteria are met and no exclusion criteria are present, consent for study participation can be obtained, and randomization can be performed.

At the baseline visit, a slit lamp examination and photo documentation and LaserFlareCellMeter (if available), corneal tomography, and Anterior Segment Optical Coherence Tomography (AS-OCT) measurements will be performed. In addition, visual acuity and vision-related quality of life will be assessed. Concomitant medication will be documented. Postoperatively, follow-up assessments will be performed at 3, 6, 12, 18, and 24 months for all subjects (postoperative visits at these time points are standard care). A slit lamp examination, concomitant medication, Adverse Events (AE), and photo documentation, as well as LaserFlareCellMeter (if available), corneal tomography, AS-OCT, and corneal endothelial cell count measurements will be performed. In addition, visual acuity and vision-related quality of life will be assessed.

The Principal Investigators will oversee and coordinate the collection, entry, and protection of data in the trial. The trial staff will collect trial-specific data using designated source documents and follow standard Good Clinical Practice (GCP) procedures to ensure accurate and consistent data collection. Any corrections made to source documentation must be documented and leave the original entries legible and signed or initialed with the correction date. A trackable audit trail is required if electronic patient files are used as source documents. All trial data must be verifiable to the source documentation and kept in a locked facility at the clinical site. Source documents include IC Forms, laboratory results, lists of adverse events and concomitant medication, NEI-VFQ-25, and documentation of existing conditions [19, 20]. Medical records will be archived according to local regulations. The Principal Investigators or their designees will document all relevant trial data in electronic case report forms (eCRFs), which will be reviewed by the clinical team for accuracy and completeness. The Principal Investigators may authorize trial staff members to sign the eCRFs to confirm data accuracy, and they will perform the final sign-off after follow-up visits.

Serious Adverse Events and Device Deficiencies must be entered in eCRF within two Business Days at the latest.

Previously determined staff ophthalmologists will decide whether CoNV sufficiently regressed following the first study intervention. Later, these data will also be centrally analyzed by graders of the CORIC and will be employed to diagnose rejection episodes and graft failure.

#### Plans to promote participant retention and complete follow-up {18b}

The distinguishing intervention between the two study arms (CXL) will be performed after trial entry. All trial participants will be monitored closely by the study team. PKPL will be the same for both groups. Follow-up is usually excellent after transplant surgery with low loss-to-follow-up rates. These patients have a long medical/ ophthalmological history and generally only participate in clinical trials if they are highly motivated. Our patients attend regular follow-up visits, especially outpatient clinics anyway. We do not foresee any difficulties in completing the evaluation. Attrition bias will be minimized by sending timely reminders of follow-up appointments to patients and arranging substitutes for any missed visits. Patients do not need to adhere to any other specific tasks.

### Data management {19}

Trial-specific data will be collected by the clinical trial staff using designated source documents. Standard GCP procedures will be followed to ensure accurate, reliable, and consistent data collection. Data will be handled pseudonymously.

The allocation sequence will be concealed from the investigators managing or assessing the study participants. After the allocation, trial participants, care providers, and outcome assessors will remain blinded throughout the trial as far as possible, except for the surgery.

The following demographic data will be collected upon inclusion: sex, age, visual acuity, intraocular pressure, study eye, uni-/bilaterality of the disease, genesis of neovascularization, duration of neovascularization, once or repeated CXL, percentage of reduction of neovascularization, fine needle diathermy, other relevant medical history, HLA type.

The CTCC will supply the IT infrastructure and data management staff. The trial database will be developed and validated before data entry based on standard operating procedures at the CTCC. The data management system is based on commercial trial software and stores the data in a database. All changes made to the data are documented in an audit trail. The trial software has a user and role concept that can be adjusted on a trial-specific basis. The database is integrated into a general IT infrastructure and safety concept with a firewall and backup system. The data are backed up daily. After completion and cleaning of data, the database is locked, and the data is exported for statistical analysis.

The clinical trial data will be entered online at the trial sites via the Internet. Plausibility checks are run during data entry, thereby detecting any discrepancies immediately. The CTCC Data Management will conduct further checks for completeness and plausibility and clarify questions with the trial sites electronically via the trial software. The trial site must answer these electronic queries without unreasonable delay. Further details will be specified in the data management plan. A guidance document and web-based training for data entry in the eCRF will be provided to the trial sites.

### Confidentiality {27}

All investigational materials and collected data will be pseudonymized using unique alphanumerical codes for each trial participant, and all applicable provisions of the data protection legislation will be followed. As part of the IC process, potential trial participants will be informed about the requirement to agree to handling pseudonymized data under applicable legislation to be eligible to participate in the trial. Patients who disagree with the data handling described in the IC form will not be enrolled in the trial. Patients will be referred to exclusively by their participant numbers. Clinical information, IC, and eCRFs will be stored closed off and protected against outside influence.

The sponsor (University of Cologne) is responsible for data processing. The sponsor representative is the Coordinating investigator.

### Plans for collection, laboratory evaluation, and storage of biological specimens for genetic or molecular analysis in this trial/future use {33}

The ophthalmologic pathology department will routinely evaluate all corneal specimens obtained during surgery. Additionally, tissue may be stored, e.g., in the blood bank of Cologne University Medical Faculty (using a separate consent form). So far, there is no study-specific storage planned. In case of any changes to the study plan, renewed ethical approval is required.

### Statistical methods

#### Statistical methods for primary and secondary outcomes {20a}

The primary analysis will be performed in the modified intention-to-treat population. The primary endpoint is the time from surgery to the first episode of graft rejection. Treatment groups will be compared using Kaplan-Meier analysis and stratified log-rank test (stratified by the number of affected corneal quadrants at baseline), counting any graft rejection episode and censoring at the last follow-up examination (or at 24 months, respectively). In addition, Kaplan-Meier analysis and log-rank test will be done in the PP population (sensitivity analysis).

As a secondary analysis, multivariable Cox proportional hazards regression will be used to assess and adjust for the effect of various risk factors and/or side effects (HLA-typing, systemic immunosuppression for other diseases, vaccination).

The secondary outcomes (Table 3), overall functional graft survival rate, and rejection-related graft failure rates will be compared between treatment groups using methods analogous to the primary endpoint. characterized descriptive statistics and boxplots per treatment group at time point CoNV will be characterized using descriptive statistics and boxplots per treatment group at each point in time. A frequency table will present the number of required CXL procedures and recurrence after transplantation in the intervention group. Best corrected visual acuity (BCVA) and vision-related quality of life scores (overall score as measured by NEI-VFQ25) will be compared using graphical methods (scatterplot, boxplot) and distribution-free tests (Mann-Whitney) [19, 20].

**Table 3:** Specification of secondary endpoints

Outcome	Specific measurement variable	Analysis metric (participant level)	Method of aggregation (summary measure for each study group)	Measurement time or time point
Overall functional graft survival rate	Graft survival	Time from surgery to graft failure or to the last observation	Rate derived from survival curve	Within 24 months after transplantation
Rejection-related graft failure rate	Rejection-related graft failure with endothelial graft rejection	Time from surgery to rejection-related graft failure or to last observation	Rate derived from survival curve	Within 24 months after transplantation
Sufficient regression of CoNV after 1st CXL and only after 2nd CXL	Regression of CoNV > 50% (intervention arm only)	Value (yes/no)	Proportion	After 1st CXL and after 2nd CXL
Recurrence of CoNV	Recurrence (after sufficient regression was reached; intervention arm only)	Value (yes/no)	Proportion	After CXL (until transplantation) and after transplantation (within 24 months)
Visual acuity (BCVA)	ETDRS charts (transformed to logMAR)	Value	Mean	Prior to CXL, prior to transplantation, and 3, 6, 12, 18, and 24 months after transplantation
Vision-related quality of life	Overall score of NEI-VFQ25	Value	Mean	Prior to CXL, prior to transplantation, and 3, 6, 12, 18, and 24 months after transplantation
Infections	Active infectious keratitis or corneal ulceration	Value (yes/no)	Proportion	Within 24 months
Delayed wound healing	Delayed wound healing: corneal epithelium not closed (delay) or closed (no delay)	Value (yes/no)	Proportion	Within 24 months after transplantation
Graft dehiscence	Graft dehiscence characterized by leakage of aqueous humor	Value (yes/no)	Proportion	Within 24 months after transplantation

Survival analysis techniques will analyze primary and key secondary endpoints, allowing all patients to contribute information for however long they were followed up. The calculation of the scales of the NEI-VFQ follows the respective manual; missing values will be handled as described therein [19, 20].

The safety analysis includes calculating and comparing specified complication rates (see secondary endpoints), adverse events, and device deficiencies. Furthermore, descriptive methods are used to assess the homogeneity of the treatment groups. Analyses will be done using SAS (SAS Institute Inc., Cary, NC, USA) or IBM SPSS Statistics (IBM, Armonk, NY, USA).

Subgroup analyses will be done based on age at transplantation, sex, pretransplantation degree of CoNV, number of previous grafts, and presence of systemic immunosuppression.

#### Interim analyses {21b}

No interim analyses are planned.

#### Methods for additional analyses (e.g., subgroup analyses) {20b}

Subgroup analyses will be done based on age at transplantation (≤ 40 years vs. > 40 years), sex, pretransplantation degree of CoNV (0–1 quadrant vs. more than 1 quadrant), number of previous grafts (0 vs ≥ 1), presence of systemic immunosuppression (yes vs. no). The expected gender distribution is 50–50, as no known gender-related factors affect CoNV, efficacy, or safety of the CXL procedure, or transplantation-associated parameters.

#### Methods in analysis to handle protocol non-adherence and any statistical methods to handle missing data {20c}

In general, missing data will not be imputed. Unless indicated otherwise, summary statistics will be reported for observed data only. The number of missing values will be given. Survival analysis techniques will analyze primary and key secondary endpoints, allowing all patients to contribute information for however long they were followed up. In calculating the scales of the NEI-VFQ, missing values will be handled as described in the manual [19, 20].

### Analysis populations

The primary analysis set is the modified intention-to-treat analysis population, which includes all randomized patients who gave IC regardless of conformance to further inclusion/exclusion criteria, performed baseline assessment, and received surgery. Patients in the assigned treatment group will be analyzed irrespective of successful CXL.

A per-protocol (PP) population will be analyzed, excluding patients who do not fulfill major inclusion criteria or have other major protocol violations. Before the final analysis, the lead investigator (or representative) will blindly review all protocol violations.

The safety population includes all randomized patients with baseline assessments (this includes patients who did not receive surgery). The analysis is according to the treatment received.

### Plans to give access to the full protocol, participant-level data, and statistical code {31c}

The datasets analyzed during the current study are available from the corresponding author on reasonable request, as is the full protocol.

### Oversight and monitoring

#### Composition of the coordinating center and trial steering committee {5d}

The sponsor is responsible for financing, conducting, and supervising of the trial, including quality management, trial design, data handling, investigator selection, and data analysis. Administrative trial project management, data management, monitoring, and safety management are done by the Clinical Trials Center Cologne.

To ensure patient safety and data validity and that the trial is conducted according to the trial protocol, as well as the principles of GCP and local legislation, all trial sites will be monitored regularly.

In addition, various committees are set up to monitor the trial. A Steering Board (SB) will continuously monitor the study’s progress and take, for example, majority decisions on all or selected questions. A Scientific Advisory Board (SAB) will provide the SB outside scientific and subject perspective advice and monitor the trial's progress. SB meets monthly, and the SAB yearly.

#### Composition of the data monitoring committee, its role, and reporting structure {21a}

A Data Monitoring and Safety Board (DSMB) of independent experts was set up. It consists of two physicians and a statistician who are not involved in the conduct of the study and are independent of the sponsor. The task of the DSMB is to monitor the safety of the study patients in the clinical trial by periodically (yearly) assessing the safety of the trial intervention and monitoring the integrity and validity of the data collected and the conduct of the clinical study following the trial protocol, the principles of GCP and local legislation. Details are provided in a DSMB charter.

A monitoring visit report is prepared for each visit, describing the progress of the clinical study and any problems. The principal coordinating Investigator (PCI) and the local principal Investigator (PI) will reasonably consider the corrective and preventive measures suggested.

#### Adverse event reporting and harms {22}

During the trial, AEs and serious adverse events (SAE) will be documented for each subject from the time IC is given until the end of the trial. Device deficiencies (DD) will be documented from CXL until pKPL.

The sponsor ensures that all persons involved in treating study patients are adequately informed of the responsibilities and actions required when AEs occur. Study patients will be asked whether they have experienced AEs or SAEs at each visit. AEs, SAEs, and DDs that occurred in the documentation period will be documented in the study patient’s medical records and the eCRF, including all information listed AE verbatim, the type of event (serious or not), beginning and end, severity, causal relationship with ID/ medical procedure, action taken and the outcome.

Regardless of whether a causal relationship between the AE and ID is suspected, study patients who develop adverse events must be monitored until all symptoms have subsided, pathological laboratory values have returned to pre-event levels, a plausible explanation is found for the AE, the study patient has died, or the study has been terminated for the study patient concerned.

A persistent AE extends continuously, without resolution, between patient evaluation time points. Such events should only be recorded once on the AE eCRF. The event's initial severity (intensity or grade) will be recorded when the event is first reported. If a persistent adverse event becomes more severe, the extreme severity should be recorded on the AE eCRF.

### Frequency and plans for auditing trial conduct {23}

Various boards will regularly monitor the study. A Steering Board will meet every 4 to 8 weeks and continuously assess overall recruitment, outcome parameters, and adverse events.

A Data Safety and Monitoring Board (DSMB) advises and supports the sponsor in evaluating the safety and efficacy of the clinical trial for patients. In addition, the DSMB recommends continuation, modification, or early termination of the clinical trial according to the criteria established by the sponsor. The DSMB will meet at least once a year.

A Scientific Advisory Board (SAB) advises the sponsor from an external, independent scientific and patient perspective and monitors the trial’s progress. The SAB meets once a year during the clinical trial's conduct.

### Plans for communicating important protocol amendments to relevant parties (e.g., trial participants, ethical committees) {25}

Changes to the study protocol may only be implemented if the sponsor, the sponsor’s representative, the PCI, and the statistician agree. Any changes to the study procedures must be made in writing and documented with reasons and signed by the sponsor’s representative, the PCI, and the statistician. Significant changes will be implemented only after the favorable opinion of the ethics committee (EC). Exceptions to this are amendments made to avoid immediate dangers. Afterwards, the sponsor will provide the trial sites with an updated version of all documents affected by the amendment. The principal investigator or sponsor provides reports, updates, and other information (e.g., safety reports and amendments) to the EC following institutional procedures.

### Dissemination plans {31a}

Following the recommendations of ICMJE, the study was registered in a public register (ClinicalTrials.gov: NCT05870566. Registered on 22 May 2023).

The EC will be informed within 90 days after the end of the study. Within one year after the end of the study, the EC will be supplied with the summary of the final study report. Study results will be published in a scientific journal and presented at congresses in mutual agreement with the sponsor. The sponsor intends to publish the clinical study results with all study sites as one group. Any publication will follow the ‘Uniform requirements for manuscripts submitted to biomedical journals (ICMJE 1997) [22].

Any publication will observe privacy concerning study patients and principal investigators. Publications will not disclose success rates or individual findings of individual study sites.

The sponsor must approve publications of study results in advance, and the sponsor reserves the right to review and comment on such documentation before publication. However, the sponsor will not withhold publication for non-scientific reasons.

By signing the contract to participate in this study, the PI declares that he or she agrees to submit the study results to the respective EC. At the same time, the principal investigator agrees that his or her name, address, qualifications, and details of his or her involvement in the clinical study may be disclosed to these bodies.

Further information including a graphical flow chart also for patients is available on the website of our department https://augenklinik.uk-koeln.de/forschung/weitere-koordinierte-programme/corneales-crosslinking/.

In addition, the patient is handed out a visit schedule at screening.

## Discussion

Corneal transplantation in high-risk patients lacks an evidence-based, simple approach to reduce rejection rates and improve graft survival. This is relevant in industrialized countries, with about 10% of all grafts being “high-risk.” It is an even greater “unmet medical need” in so-called lower to middle-income countries (LMIC) worldwide, where > 75% of all transplants are “high-risk.”

Previous preclinical and early clinical studies have demonstrated the efficacy of (lymph)angioregression (and subsequently improved graft survival) achieved through CXL or FND [23, 24]. Registry studies [8], meta-analysis [6], preclinical investigations [10, 11, 13, 25, 26], and small retrospective clinical studies [24] provide additional evidence for successful (lymph)angioregression improving subsequent corneal graft survival.

This multicenter-controlled IIT aims to confirm these findings in a multicenter randomized controlled prospective trial for the first time. We will examine the effectiveness and potential adverse effects of pretransplant regression of pathological CoNV in high-risk eyes using CXL (and FND if additionally needed). While CXL is primarily used to stabilize the cornea in cases of ectasia, its potential application as a (lymph)angioregressive treatment in patients with CoNV has been described [14]. Previous small-scale experimental and clinical studies have demonstrated the tolerability of CXL treatment with no significant intraoperative or postoperative complications. Epithelial closure and suturing of donor corneas into previously crosslinked host corneas was uneventful [14, 27].

This study aims to test CXL as a safe clinical adjunctive treatment before high-risk pKPL to promote graft survival. This is a more cost-effective and ethically justifiable approach than repeated corneal transplants following rejection. Costs are particularly important in LMIC worldwide, where most affected patients live, and the scarcity of transplant corneas makes careful use mandatory [5].

If successful, a confirmatory clinical trial will follow. This novel approach will significantly contribute to patients’ quality of life worldwide and the care of patients who must undergo high-risk corneal transplantation.

### Trial status

Version of 15.09.2023, Version V03_0

The trial has formally started in August 2022, recruitment has started in December 2023. Recruitment is planned to be completed by Q III 2025. The study runs until 2027. Funding is provided for the entire trial period until July 2027.

## Abbreviations

AE: Adverse event
AS-OCT: Anterior segment optical coherence tomography
BCVA: Best corrected visual acuity
BMBF: Bundesministerium für Bildung und Forschung
CoNV: Corneal neovascularization
CORIC: Cologne Ophthalmological Reading and Image Analysis Center
CRF: Case report form
CXL: Corneal crosslinking
DD: Device deficiency
DSMB: Data Safety and Monitoring Board
EC: Ethics committee
EOT: End of trial
ETDRS: Early treatment diabetic retinopathy study
EU: European Union
FND: Fine needle diathermy
FPFV: First patient first visit
FU: Follow-up
GCP: Good clinical practice
HLA: Human leukocyte antigen
IC: Informed consent
IIT: Investigator initiated trial
IMSB: Institute of Medical Statistics und Computational Biology
ISO: International Organization for Standardization
LPFV: Last patient first visit
LPLV: Last patient last visit
mITT: Modified Intention-to-treat
NEI-VFQ: National Eye Institut Visual function questionnaire
PCI: Principal Coordinating Investigator
LKP: Leiter der klinischen Prüfung
PI: Principal investigator
pKPL: Penetrating keratoplasty
PP: Per-protocol
SAB: Scientific Advisory Board
SAE: Serious adverse event
SB: Steering Board
UV: Ultraviolet radiation
VEGF: Vascular endothelial growth factor
